# Meta-Analysis and Systematic Review of the Thermal Stress Response: *Gallus gallus domesticus* Show Low Immune Responses During Heat Stress

**DOI:** 10.3389/fphys.2022.809648

**Published:** 2022-01-28

**Authors:** Sharif Hasan Siddiqui, Mousumee Khan, Darae Kang, Hyun Woo Choi, Kwanseob Shim

**Affiliations:** ^1^Department of Animal Biotechnology, College of Agriculture and Life Sciences, Jeonbuk National University, Jeonju, South Korea; ^2^Department of Biomedical Sciences and Institute for Medical Science, Jeonbuk National University Medical School, Jeonju, South Korea; ^3^Department of Animal Science, College of Agriculture and Life Sciences, Jeonbuk National University, Jeonju, South Korea; ^4^Department of Agricultural Convergence Technology, College of Agriculture and Life Sciences, Jeonbuk National University, Jeonju, South Korea

**Keywords:** heat stress, broiler, weight gain, immunoglobulin, mortality

## Abstract

Heat stress, which affects broiler growth performance and immunity, is a major concern in the poultry industry. This meta-analysis aimed to demonstrate the significant effect of heat stress on broiler mass gain and immunoglobulin levels, which regulates the mortality rate of broilers. A total of 2,585 studies were downloaded from PubMed, Web of Science, and Google Scholar from January 1, 2015, to September 1, 2021. Eventually, 28 studies were selected based on specific criteria. The results for body mass gain, total mass of immune organs (thymus, spleen, and bursa of Fabricius), immunoglobulin (IgA, IgG, and IgM) levels, and mortality rate were analyzed using odds ratio or the random-effects model (REM) at a confidence interval (CI) of 95%. Compared to the control, heat stress significantly decreased body mass gain (10 trials: REM = 1.35, 95% CI: 1.21, 1.50). Compared to that in the control, heat stress significantly increased immunoglobulin levels: IgA (7 trials: REM = 1.69, 95% CI: 0.90, 3.16), IgG (6 trials: REM = 1.24, 95% CI: 0.85, 1.81), IgM (8 trials: REM = 0.69, 95% CI: 0.44, 1.08), and heat stress also increased the broiler mortality rate (6 trials: REM = 0.06, 95% CI: 0.01, 0.27). However, there were no significant changes in the immune organs between the control and heat-stressed groups. In conclusion, heat stress remarkably alters the mass gain and immunoglobulin levels of broilers, which may be a cause of the high mortality rate.

## Introduction

Heat stress is a major concern in this era of global warming, especially in tropical and subtropical regions (Sun et al., 2019). Heat stress is experienced when high temperature prevails where the animals perceive discomfort. Heat stress occurs at the temperature at which an animal cannot dissipate its entire body temperature to the surrounding environment (Sejian et al., 2018). It has been reported that heat stress plays a negative role in animal welfare and animal production, especially in broilers (Soravia et al., 2021). During heat stress, energy metabolism is reduced, leading to poor organ development and decreased mass gain (Fausnacht et al., 2021). It has been reported that energy metabolism and immunity are related (Wang et al., 2020). This relationship indicates that low energy metabolism is one of the crucial factors for low immune function (Ganeshan and Chawla, 2014).

Immunity is a state or condition that prevents diseases associated with the development of pathogenic microorganisms (Zheng et al., 2020). The adrenal cortex is activated by heat stress and secretes corticosterone, which affects the immune system (Smith and Vale, 2006). However, a robust immune system plays a pivotal role in animal production, especially in broiler production (Siddiqui et al., 2020a; Song et al., 2021). In contrast, a poor immune system has a negative effect on animal growth performance and mortality (Doeschl-Wilson et al., 2009). Moreover, immunoglobulins regulate immune functions and elicit immune responses (Ulfman et al., 2018). Again, the levels of the immunoglobulin are altered by heat stress (Calefi et al., 2016). This information reveals that there is a relationship between heat stress and immune response.

Immunoglobulins are heterodimeric proteins that contain two heavy and two light chains characterized by variable and constant domains that bind to antigens and Fc receptors, respectively (Schroeder and Cavacini, 2010). It is well known that a high number of IgG, IgM, and IgA immunoglobulins are found in chickens, and the principals of these immunoglobulins are like mammalian' immunoglobulins respectively (Ayaz et al., 2008). Moreover, immunoglobulins are known as antibodies, which indicate the immune status through antigen recognition and binding (Sela-Culang et al., 2013). It is well known that the broiler mortality rate depends on many factors; among them, antibody levels are a crucial factor for mortality during heat stress (Wasti et al., 2020). Therefore, we hypothesize that the immunoglobulin level can be used as an indicator of poultry growth performance.

There are many studies that have revealed the effect of heat stress on broiler growth performance, including mass gain, feed intake, and feed conversion ratio (He et al., 2020; Emami et al., 2021). Broilers are highly heat stress-sensitive (Nassar and Elsherif, 2018) and lose their immune system function, which leads to death (Hirakawa et al., 2020). However, to the best of our knowledge, no meta-analysis has focused on the immunity level of broilers exposed to heat stress. Therefore, our study was conducted to find out a relation between heat stress and broiler's immunoglobulin level through a systematic review and meta-analysis of published research data.

## Methodology

### Methods

This study was conducted according to the Preferred Reporting Items for Systematic Reviews and Meta-Analyses (PRISMA) criteria (Moher et al., 2009). This study was categorized into two groups based on temperature level for meta-analysis.

### Search Strategy

Keywords for our study were searched using three electronic databases, including PubMed (National Library of Medicine, Bethesda, Maryland, USA), Web of Science (Thomson Reuters, London, UK), and Google Scholar, beginning January 1, 2015 and concluding September 1, 2021. The criteria for the finalized studies were that it should have been performed with different strains of broiler chickens (Table 1) and published as an original full article in a peer-reviewed journal. The following key terms for literature exploration were used for the different searches: heat stress, thermal condition, hot environment, high temperature, broiler, chicken, poultry, growth performance, mass gain, mortality, thymus, spleen, bursa of Fabricius, immunity, IgG, IgA, and IgM. The title and abstract of the studies were identified by searching keywords following the selection criteria.

**Table 1:** Characteristics of all selected studies.

**Study**	**Strain**	**No. of broilers/group**	**Age for experiment (days)**	**Thermoneutral temperature**	**Heat stress temperature**
Siddiqui et al., 2021a	Ross-308	60	21	24 ± 1 °C	34 ± 1 °C
Siddiqui et al., 2020a	Ross-308	60	21	24 ± 1 °C	34 ± 1 °C
Goo et al., 2019	Cobb	76	21	20 ± 1 °C	27 ± 1 °C
Liu et al., 2019	Huaixiang chickens	48	35	26 ± 1 °C	32 ± 1 °C
Kikusato et al., 2021	Ross-308	90	14	22 ± 2 °C	33 ± 1 °C
Abo Ghanima et al., 2019	Arbor Acres, Avian-48	60	14	25 ± 2 °C	34 ± 1 °C (from 9:00 to 18:00 h then 25 ± 2 °C)
Jiang et al., 2020	Ebayka	5	30	20 ± 2 °C	30 ± 2 °C
Ruff et al., 2020	Cobb 500	160	21	24 ± 1 °C	35 ± 1 °C
Ruff et al., 2021	Cobb 500	160	21	24 ± 1 °C	35 ± 1 °C
Chegini et al., 2019	Ross-308	Not mentioned	21	22 ± 1 °C	33 ± 1 °C (from 9:00 to 18:00 h then 25 ± 2 °C)
Attia and Hassan, 2017	Ross-308	35	28	28 ± 4 °C	36 ± 3 °C
Song et al., 2018	Arbor Acres	48	21	22 ± 1 °C	34 ± 1 °C (from 9:00 to 18:00 h then 22 ± 2 °C)
Awad et al., 2020	Cobb 500, Ross-308	Not mentioned	22	23 °C	34 °C
Honda et al., 2015	Cobb 500	24	1	30 ±1 °C	38 ± 1 °C
Calefi et al., 2016	Cobb	Not mentioned	14	Not mentioned	35 ± 1 °C
Hamidi et al., 2021	Ross 308	15	21	21 ± 1 °C	36 ± 1 °C
Hosseini-Vashan et al., 2016	Arian	88	21	21 °C	34 ± 1 °C
Hirakawa et al., 2020	Ross	Not mentioned	22	24 ± 0.5 °C	34.5 ± 0.5 °C
Alhotan et al., 2021	Ross 308	50	21	22 ±1 °C	33 ± 1 °C for 8 h from 08:00 to 16:00 and 22 ± 1°C for the remaining time per d
Zhang et al., 2018	Arbor Acres	Not mentioned	21	25 ± 1 °C.	35 ± 1 °C
He et al., 2019	Yellow-feathered	96	21	24 ± 2 °C	37 ± 2 °C for 8 h from 09:30 to 17:30 and 24 ± 2 °C for the remaining time per d
Abudabos et al., 2021	Ross 308	36	21	24 ±1 °C	35 ± 1°C
Tang et al., 2021	Ma chickens	40	21	25± 2 °C	35 ± 2°C
Saad et al., 2021	Cobb 500	30	7	22–25 °C	32–35 °C
Attia et al., 2018	Ross-308, Cobb-500	30	21	25 °C	32 °C
Zampiga et al., 2021	Ross 308	150	21	21 °C	30 °C
Beckford et al., 2020	Ross 708	80	24	21 °C−23 °C	34 °C−36 °C
Del Vesco et al., 2015	Cobb 500	30	22	21 °C	38 °C

### Selection Criteria

The literature was evaluated for its suitability and relevance with respect to the criteria described below. This selection process is graphically illustrated in Supplementary Figure 1. A study was selected for meta-analysis when it fulfilled the particular requirements below:

1. The study must contain a thermoneutral group, i.e., this condition acts as a control group compared to heat stress group.
2. The study had to be revealed in terms of at least two parts, such as the heat stress initiation point and endpoint, to analyze the effect of heat stress. Moreover, a study must analyze heat stress levels in comparison with the Control. This treatment usually affects broiler growth performance, where consistent error data were also provided. The literature was selected based on other parameters, such as the mass of the immune organ, immunoglobulin levels, and mortality.
3. All the data had to be presented as mean with standard deviation (SD) and/or standard error (SE), either in tables or figures.
4. Only self-paced studies were selected, which means those studies were accomplished appropriately by the authors and discussed their own way.
5. Only those studies that presented the feedback of heat stress on the broiler were selected, which means the selected article must have shown the response of heat stress. For instance, when the broiler faced heat stress challenge than had showed some phenotypic, genotypic, and physiochemical changes.
6. Only studies that were published in an international peer-reviewed journal were selected. That means, we did not select preprint as well as Non-published articles.

### Study Classification

A total of 28 studies that satisfied the selection criteria were selected from initial 1,770 studies identified from peer-reviewed scientific journals. The studies were characterized based on descriptive environmental or related variables evaluated by the researchers, for example, the environmental temperature during an animal trial. Two different groups were identified: (1) Control (Thermoneutral temperature) and (2) Treatment group (Heat stress group). Additionally, we selected recent and detailed information-related studies if duplicate studies were observed.

### Data Extraction

We extracted data from the selected studies independently using predesigned combined consistent reporting forms divided by the study area. The Plot Digitizer software (http://plotdigitizer.sourceforge.net/) was used to present relevant data that were not directly provided in the selected study. For accurate analysis, specific information was extracted: data source (author, publication year), population characteristics (species, age, breed, and number), research design, data collection (immunoglobulin forms, methods, and data for analysis), description, and frequency of immunity, and accuracy of immunoglobulin on immunity diagnosis (sensitivity, specificity, and corresponding receiver operating characteristic). In this manner, most variables in the accuracy analysis were also included in the selected study data for risk assessment. However, data accuracy was affected by immunity risk based on immunoglobulin variations, including a comparison of immunoglobulin level, calculation, and accustomed covariates. For analysis, the level of agreement between two variables and the interaction correlation coefficient was analyzed and interpreted according to the Kappa coefficient (Warrens, 2021). For example, to measure inter-rater reliability, we have used Kappa-coefficient, which have helped to assess the same phenomenon between the Control and Heat stress groups. The analysis showed an almost absolute correlation between the two variables (Control and Heat stress) [interaction correlation coefficient = 0.99; 95% confidence intervals (CI): 0.99 and 0.99]. Therefore, we were confident that there were minimal researcher and study biases in measurements when extracting data for meta-analysis (Landis and Koch, 1977).

### Study Quality Assessment

The methodological quality of the selected studies was quantified using the physiotherapy evidence database (PEDro) scale, although this scale is not related to the inclusion criteria. However, the PEDro scale helped to get sufficient statistical information of journal and rapidly identify the valid journal for meta-analysis (Moseley et al., 2002). The PEDro scale measures the quality of research compared with 11 criteria related to the experimental design. The scale score ranges from 0 to 10. The score for high-quality article is ≥ 7, moderate quality article is 5–6, and poor quality article is ≤ 4 (Machado et al., 2016).

### Data Analysis

R software V 4.1.0 (Vienna, Austria: R Foundation for Statistical Computing) was used to conduct the meta-analysis. To accomplish this analysis, we used the “metafor” package in R (Viechtbauer, 2010). The size effect of the incessant results was analyzed using the random-effects model (REM) with 95% CI, and dichotomous results were analyzed using the odds ratio (OR). Heterogeneity among different studies was analyzed using Cochran's Q statistic followed by the I2 test and study bias was determined using funnel plots. Considerable heterogeneity was established if the *p* = 0.05 (Q statistic) and I2 was 50, and then, the random-effects model was applied; otherwise, the fixed-effects model was considered. Following the Cochrane Handbook, the risk of publication bias was measured using Begg's test (Van Enst et al., 2014), if the number of trials was up to 10. *P*-value of 0.05 was considered as no published study bias.

## Results

### Study Retrieval and Selection

The study search and selection procedures are shown in Supplementary Figure 1. A total of 2,585 studies were collected from an electronic database. A total of 815 studies were omitted for repeated collection, and 128 studies were screened out from 1,770 studies by reading the title and abstract for lower relation, unsuitable articles such as reviews, comments, and involvement of other parameters. A total of 128 studies were fully reviewed, and 28 studies were finally selected for meta-analysis based on our research objective. A short description of the selected studies is provided in Table 1. Finally, our selected 28 studies were evaluated against the PEDro scale criteria (Supplementary Figure 2). The obtained PEDro scale of 8.1 ± 0.9 (mean ± SE) indicated that all selected studies were considered “high quality”.

### Analysis of Studies That Investigated Broiler Mass Gain and Study Biases

**Figure 1:**
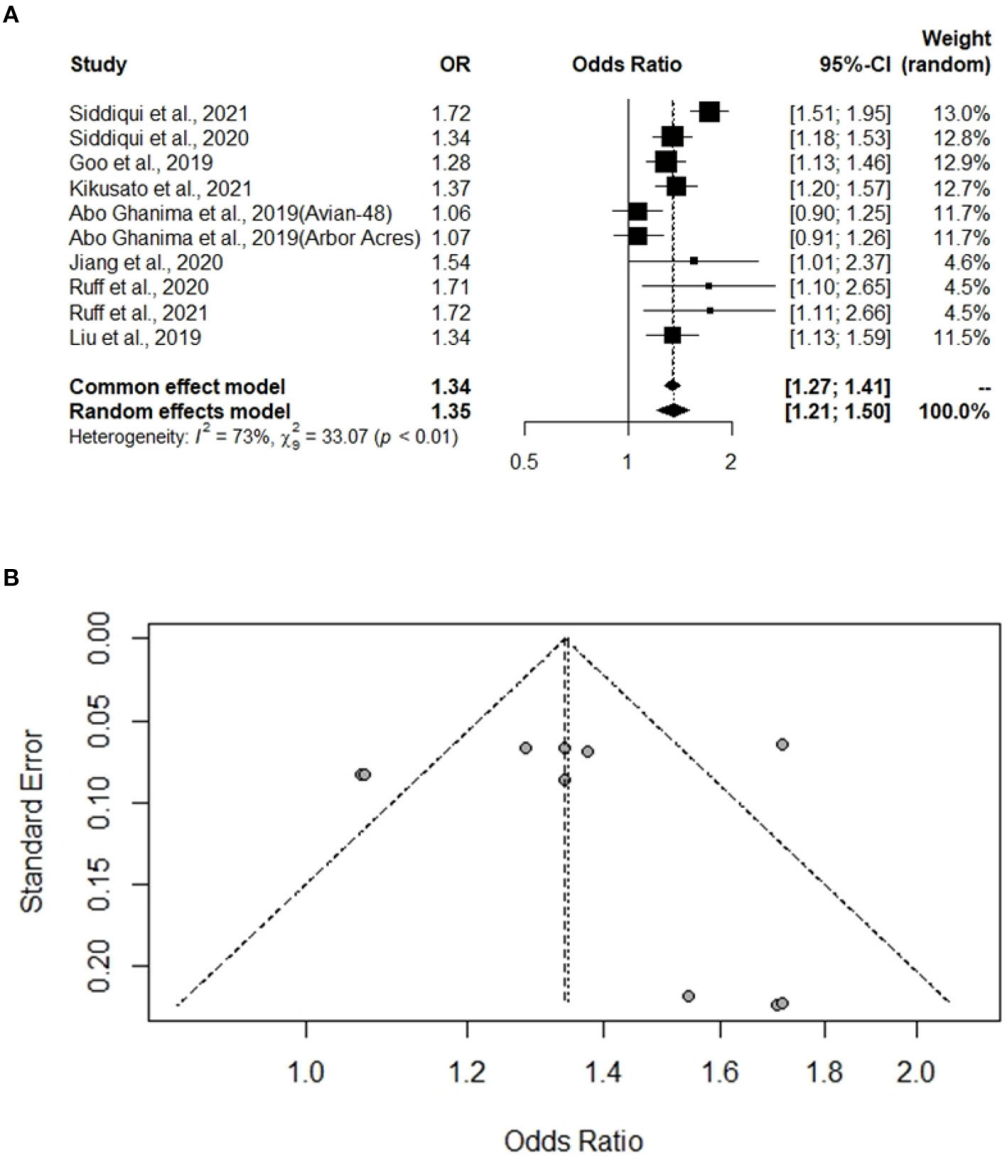
Forest plot and funnel plot representing the effect of heat stress on broiler body mass gain. **(A)** Forest plot indicates that the heat stress significantly changes the body mass gain and **(B)** asymmetric funnel plot indicates the possible biases of our selected journal.

A total of 10 experiments from nine studies (Abo Ghanima et al., 2019; Goo et al., 2019; Liu et al., 2019; Jiang et al., 2020; Ruff et al., 2020, 2021; Siddiqui et al., 2020a,b; Kikusato et al., 2021) reported a comparative body mass gain between the Heat stress and Control groups. Significant heterogeneity was found; thus, the random-effects model was applied (I^2^ = 73%, *P* < 0.01) (Figure 1A). Body mass gain was significantly decreased in the broilers exposed to Heat stress compared to that in the Control group (OR 1.35, 95% CI: 1.21, 1.50). The funnel plot represents publication bias; the funnel plot of body mass gain was asymmetrical, indicating there were biases in the selected publications (Figure 1B).

### Analysis of Studies That Investigated Immune Organ Mass and Study Biases

**Figure 2:**
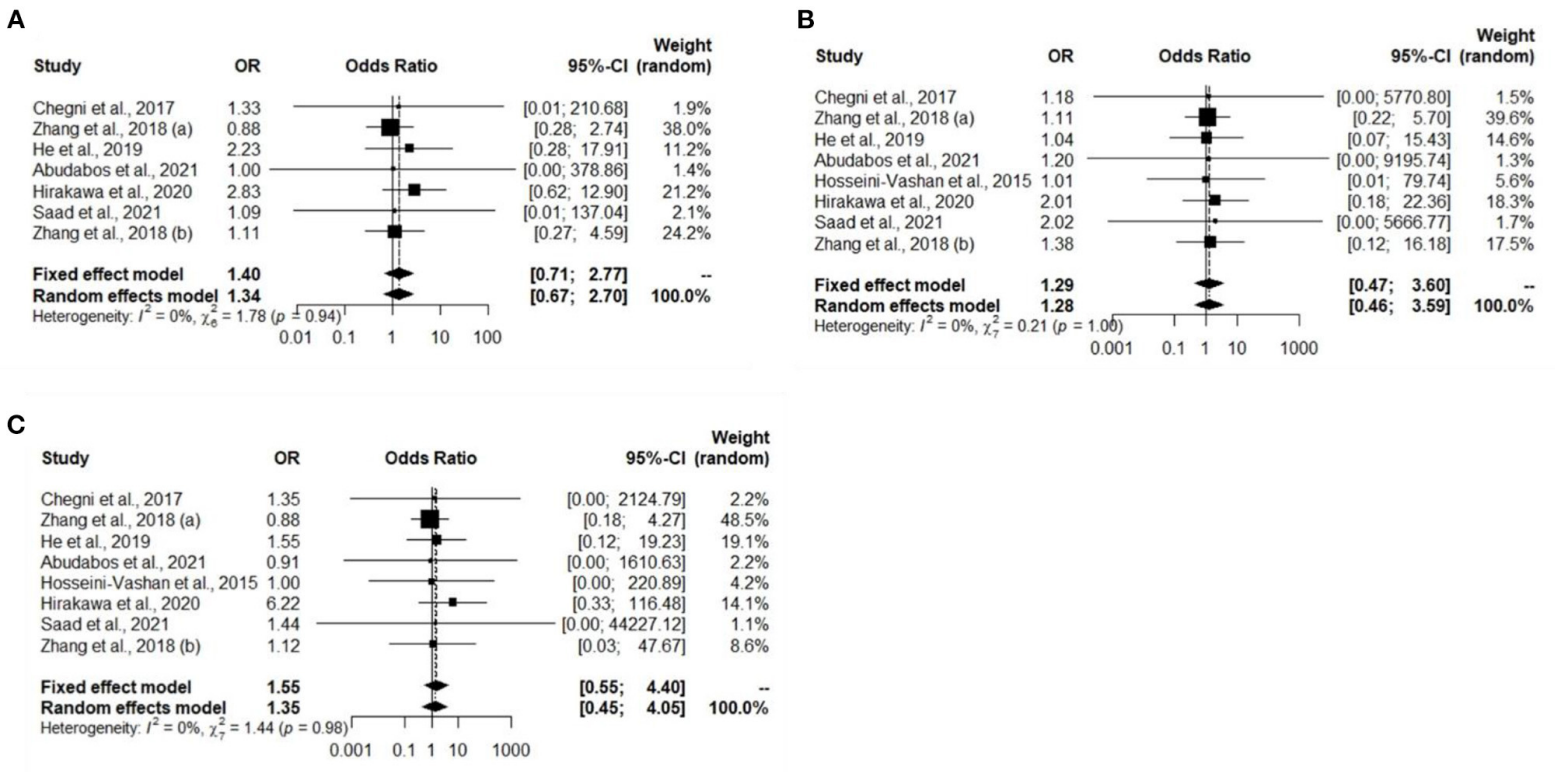
Forest plots representing the effect of heat stress on immune organ mass. **(A)** Forest plot indicates that the heat stress did not significantly change the thymus mass, **(B)** forest plot indicates that the heat stress did not significantly changes the spleen mass, and **(C)** forest plot indicates that the heat stress did not significantly change the bursa of Fabricius mass.

**Figure 3:**
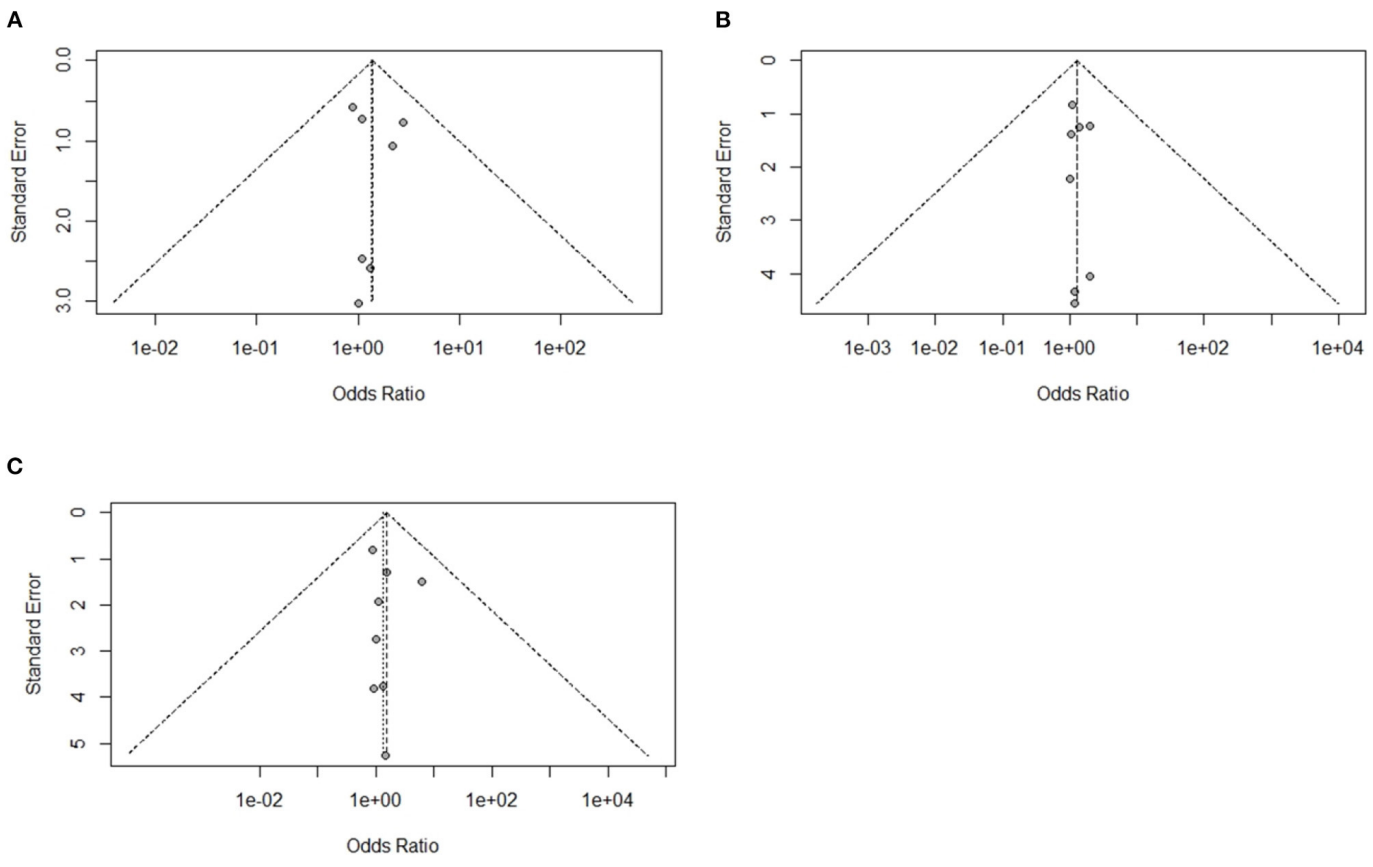
Funnel plots representing the effect of heat stress on immune organ mass journal publication bias. **(A)** Symmetric funnel plot consistent with lower likelihood of publication bias of our selected thymus related journal, **(B)** symmetric funnel plot consistent with lower likelihood of publication bias of our selected spleen related journal, and **(C)** symmetric funnel plot consistent with lower likelihood of publication bias of our selected bursa of Fabricius related journal.

A total of eight experiments from eight studies (Hosseini-Vashan et al., 2016; Zhang et al., 2018; Chegini et al., 2019; He et al., 2019; Hirakawa et al., 2020; Abudabos et al., 2021; Saad et al., 2021) reported comparative immune organ masses between the Heat stress and Control groups. Notably, no heterogeneity was found, and there was a significant difference between the Heat stress and Control groups; thus, the random-effects model was applied (I^2^ = 0%, *P* = 0.94, 1.00, 0.98) (Figures 2A–C). The immune organs (thymus, spleen, and bursa of Fabricius) were not significantly different between the Heat stress and Control groups. The study bias was analyzed using a funnel plot. None of the selected studies related to immune organs (thymus, spleen, and bursa of Fabricius) showed biases. We confirmed the absence of bias by visual inspection of the funnel plot (Figures 3A–C).

### Analysis of Studies That Investigated Immunoglobulin Levels and Study Biases

**Figure 4:**
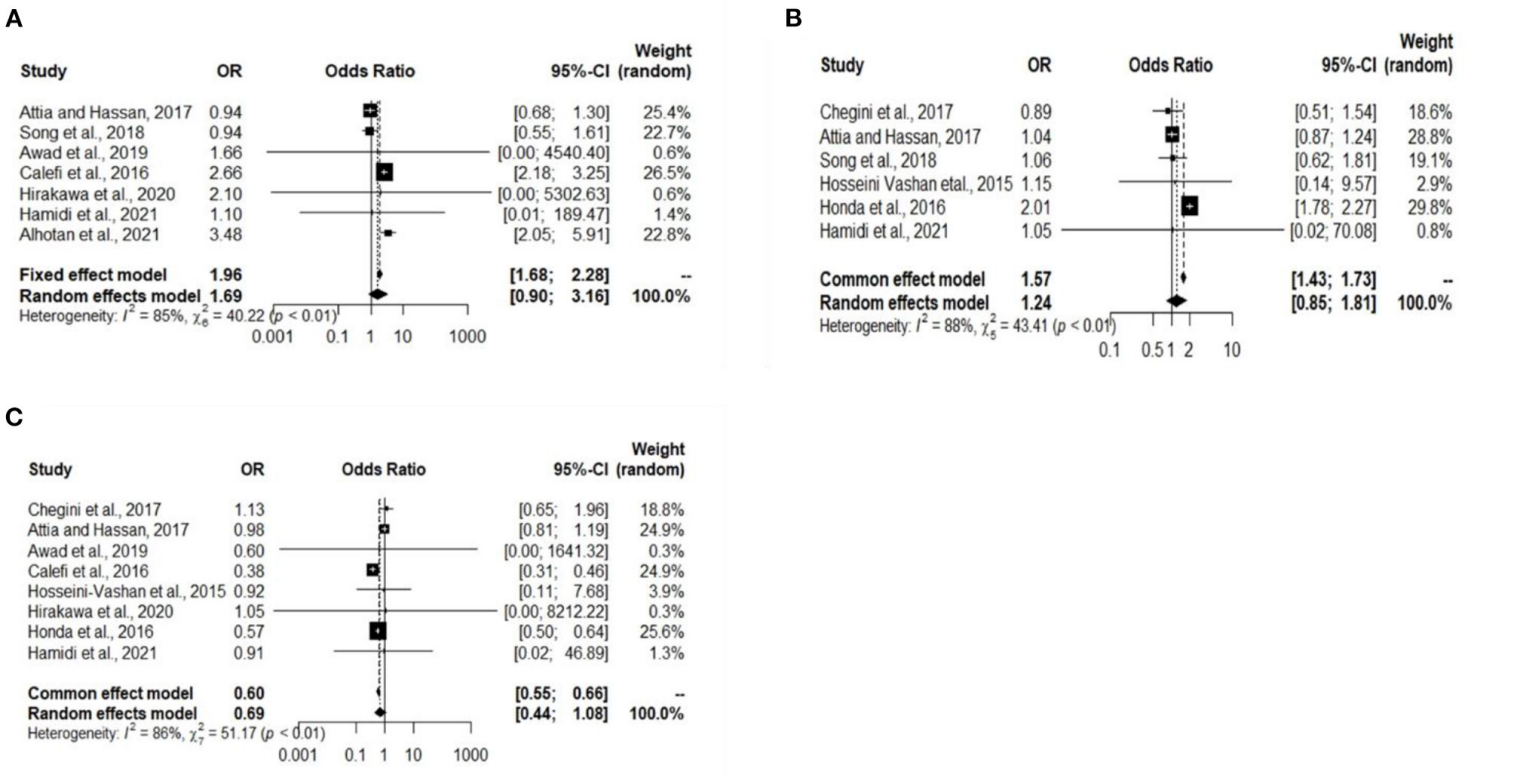
Forest plots representing the effect of heat stress on immunoglobulins level. **(A)** Forest plot indicates that the heat stress significantly changes the broiler blood IgA, **(B)** forest plot indicates that the heat stress significantly changes the broiler blood IgG, and **(C)** forest plot indicates that the heat stress significantly changes the broiler blood IgM.

**Figure 5:**
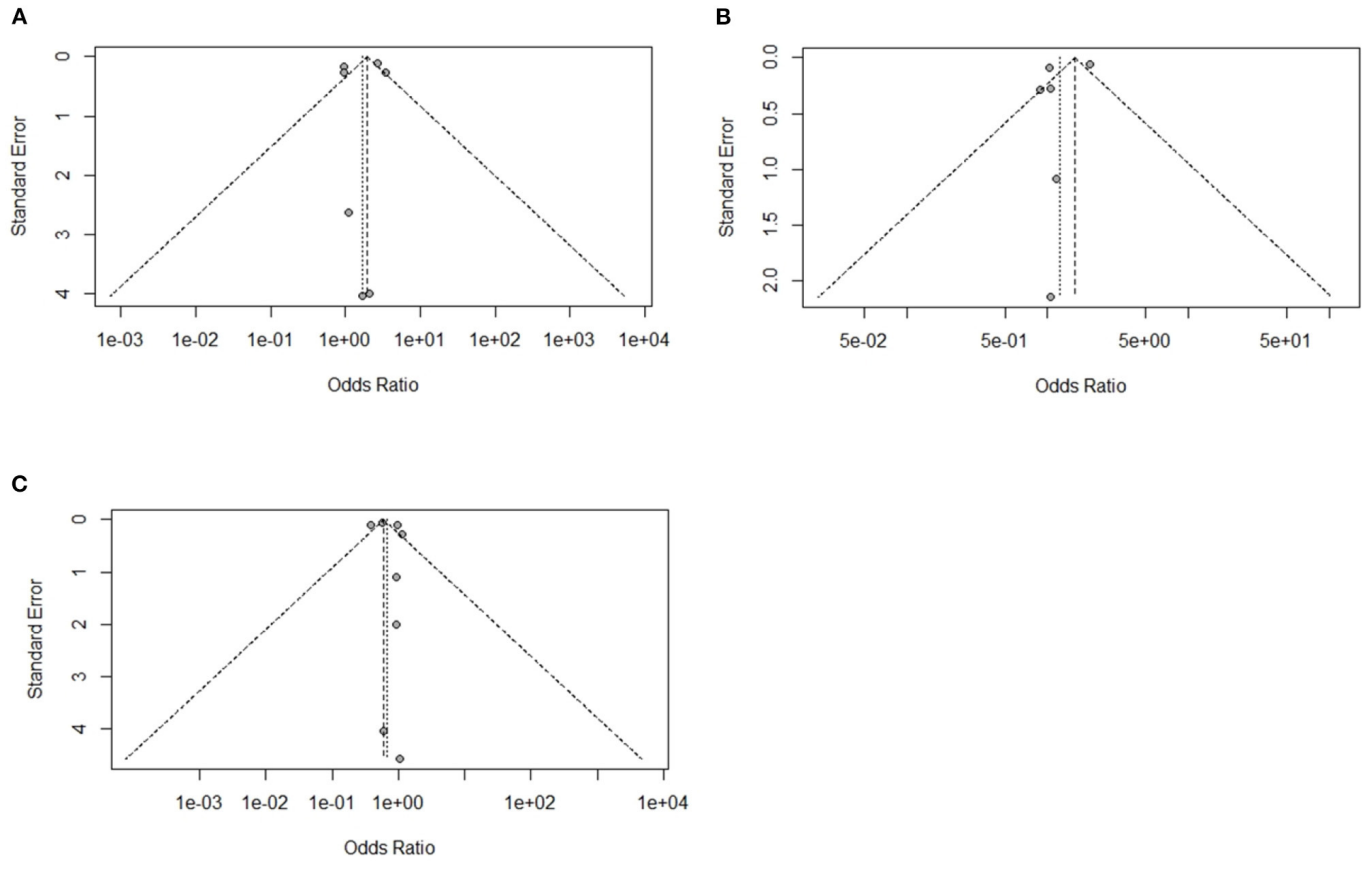
Funnel plots representing the effect of heat stress on immunity level. **(A)** Asymmetric funnel plot indicates the possible biases of our selected IgA related journal, **(B)** asymmetric funnel plot indicates the possible biases of our selected IgG related journal, and **(C)** asymmetric funnel plot indicates the possible biases of our selected IgM related journal.

A total of 10 experiments from 10 studies (Honda et al., 2015; Calefi et al., 2016; Hosseini-Vashan et al., 2016; Attia and Hassan, 2017; Song et al., 2018; Chegini et al., 2019; Awad et al., 2020; Hirakawa et al., 2020; Alhotan et al., 2021; Hamidi et al., 2021) reported comparable immunoglobulin levels (IgA, IgG, and IgM) between Heat stress and Control groups. Significant heterogeneity was found and significant difference between the Heat stress and Control groups, indicating that the random-effects model was applicable with I2 = 85%, 88%, and 86% (*P* < 0.01) for IgA, IgG, and IgM, respectively (Figures 4A–C). The IgA (OR 1.69, 95% CI: 0.90, 3.16), IgG (OR 1.24, 95% CI: 0.85, 1.81), and IgM (OR 0.69, 95% CI: 0.44, 1.08) were significantly decreased in broilers exposed to Heat stress compared to that in the Control group. Publication biases were analyzed using a funnel plot. Visual inspection confirmed that the IgA-and IgM-related studies did not have biases (Figures 5A–C).

### Analysis of Studies That Investigated Broiler Mortality and Study Biases

**Figure 6:**
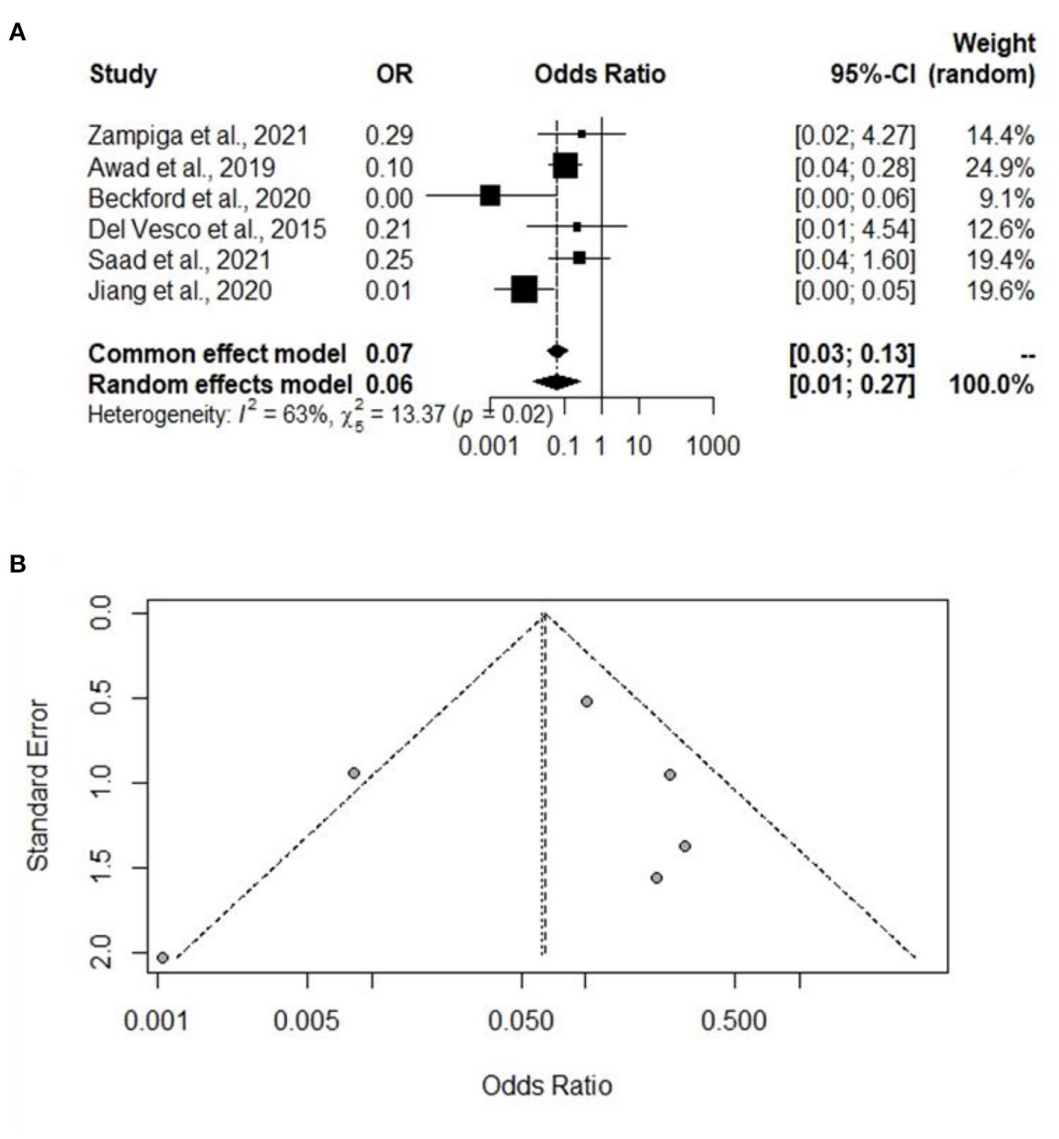
Forest plot and funnel plot representing the effect of heat stress on broiler mortality rate. **(A)** Forest plot indicates that the heat stress significantly increases the mortality rate and **(B)** symmetric funnel plot consistent with lower likelihood of publication bias of our selected journal related mortality rate.

A total of six experiments from six studies (Del Vesco et al., 2015; Awad et al., 2020; Beckford et al., 2020; Jiang et al., 2020; Saad et al., 2021; Zampiga et al., 2021) reported a comparative mortality rate between the Heat stress and Control groups. Significant heterogeneity was found, and a significant difference was found between the Heat stress and Control groups, indicating that the random-effects model is to be applied (I^2^ = 63%, *P* =0.02) (Figure 6A). The mortality rate was significantly higher in the broilers exposed to Heat stress than in the Control group (OR 0.06, 95% CI: 0.01, 0.27). Publication biases in mortality-related studies were analyzed using a funnel plot. Visual inspection of the funnel plot confirmed the absence of biases in the mortality-related studies (Figure 6B).

## Discussion

A number of review articles have been published on heat stress-related broiler performance (Liu et al., 2020; Ncho et al., 2021), laying hens robustness (Mignon-Grasteau et al., 2015), carcass traits (Piray and Foroutanifar, 2021), and free amino acid concentration (Jafari et al., 2021). However, to the best of our knowledge, this is the first meta-analysis that explores immunoglobulin levels in broilers during heat stress. In the current meta-analysis with 28 published studies, we observed that heat stress significantly decreased broiler body mass gain and immunoglobulin levels without any attendant change in immune organs (thymus, spleen, and bursa of Fabricius), but significantly increased mortality. As this meta-analysis detected moderate to high heterogeneity response traits, a random-effects model was used, as such models represent study variation except for a few selected journals (DerSimonian and Kacker, 2007).

Heat stress reduces the growth performance of broilers (Awad et al., 2020). Heat stress increases hypoxia (McBryan et al., 2013), which affects metabolism, causing an energy imbalance in the body and increasing energy expenditure (Kalia et al., 2017), thus reducing cell proliferation and viability (Siddiqui et al., 2020c,d). This phenomenon eventually decreases growth performance (Balog et al., 2000). Moreover, heat stress increases inflammation levels (Hoekstra et al., 2019). Inflammation and immunoglobulin levels have a negative relationship; a previous study reported that inflammation reduces immunoglobulin levels (Aschermann et al., 2010).

To reveal the immune function of lymphoid organs, we have checked different lymphoid organs (thymus, spleen, and bursa of Fabricius). Immunity level depends on different lymphocytes, T cells and B cells (Kato et al., 2013), that originate from different lymphoid organs (Neely and Flajnik, 2016). However, the relationship between the level of immunity and the mass of the lymphoid organ remains unclear (Shushimita et al., 2014). A previous study reported that heat stress reduces immunity without altering lymphoid organ mass (Niu et al., 2009). In this meta-analysis, heat stress did not affect the mass of the thymus, spleen, and bursa of Fabricius. However, heat stress changes immunoglobulin levels, which leads to lowered immunity.

The immune function of an animal can be altered by different factors; among which, environmental ones, such as heat stress, are crucial. The glucocorticoid hormone (corticosterone) is increased in the blood because of heat stress, which acts as a crucial factor for suppressing the induction of inflammation (Bagath et al., 2019). However, our previous study showed that a high dose of exogenous glucocorticoids causes toxicity and inhibits cell viability and proliferation (Siddiqui et al., 2021b). Immunity and immunoglobulin levels have a positive relationship. A previous study reported that immunoglobulins act as an important enhancer for the immune function of animals (Ulfman et al., 2018). In contrast, heat stress increases immunoglobulin levels in animal blood (Svobodová et al., 2014). Therefore, IgA and IgM levels increase in heat-stressed broiler chickens (Honda et al., 2015). Moreover, IgG levels also increase during heat stress, which indicates the immunity level (Filipe et al., 2012). Nonetheless, heat stress promotes the mortality rate by damaging protein structure (Hasan Siddiqui et al., 2020) decreasing immune function (Li et al., 2020). This meta-analysis also showed that the mortality rate of broilers was high in the Heat stress group with high heterogeneity.

This meta-analysis had certain limitations. First, owing to the small number of selected studies, we were unable to conduct an analysis based on broiler sex, strain, heat stress temperature, and age. Second, potential biases might be consequences of methodological issues in some studies. Third, significant heterogeneity was found in the endpoints that may have been caused by different temperatures and ages in each study.

## Conclusion

In conclusion, this meta-analysis focused on the negative effects of heat stress on broiler body mass gain, immunoglobulin levels, and mortality. This study highlights the importance of heat stress in the poultry industry to develop strategies that reduce stress levels and prevent broiler production losses.

## Data Availability Statement

The original contributions presented in the study are included in the article/Supplementary Material, further inquiries can be directed to the corresponding author.

## Author Contributions

SHS and KS conceived and designed the study. SHS extracted information and analyzed the data. SHS and MK wrote the manuscript. KS, DK, and HC reviewed the manuscript. All authors approved the final manuscript for submission.

## Funding

This work was supported by the Basic Science Research Program through the National Research Foundation of Korea (NRF) funded by the Ministry of Education (Project No. 2020R1I1A3A04038058).

## Conflict of Interest

The authors declare that the research was conducted in the absence of any commercial or financial relationships that could be construed as a potential conflict of interest.

## Publisher's Note

All claims expressed in this article are solely those of the authors and do not necessarily represent those of their affiliated organizations, or those of the publisher, the editors and the reviewers. Any product that may be evaluated in this article, or claim that may be made by its manufacturer, is not guaranteed or endorsed by the publisher.
