# Supplementary material for: Meta-Analysis and Systematic Review of the Thermal Stress Response: Gallus gallus domesticus Show Low Immune Responses During Heat Stress
Source: Front Physiol. 2022 Jan 28;13:809648. doi: 10.3389/fphys.2022.809648 (PMC8832064; doi:10.3389/fphys.2022.809648)
Supplement: Supplementary file 1 [file Data_Sheet_1.docx]

Supplementary Material


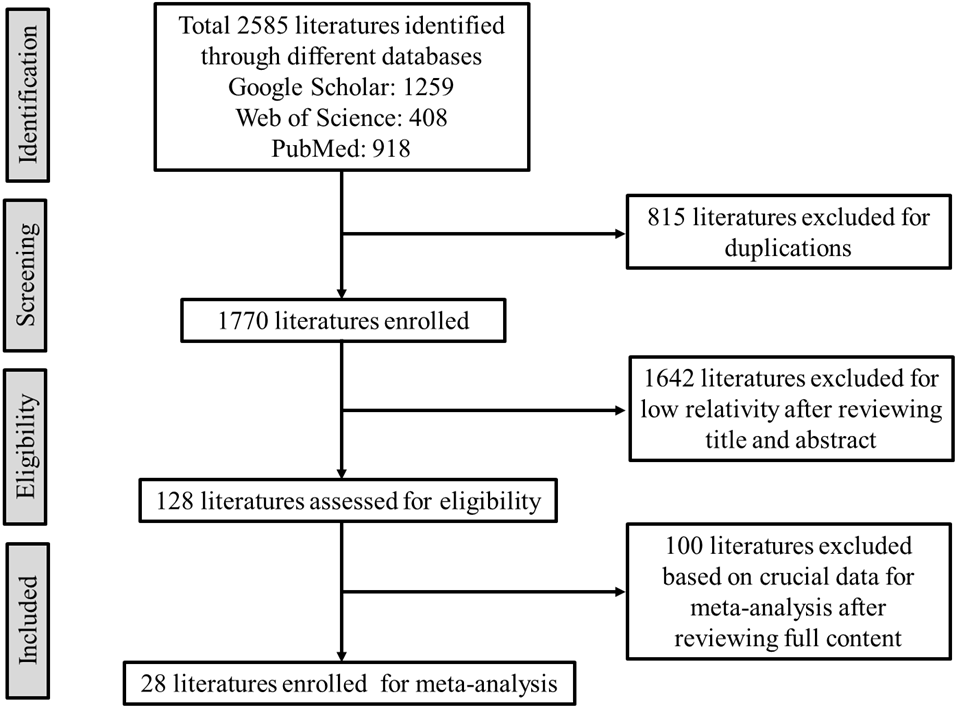


**Supplementary Figure 1.** The study selection flowchart. Randomized controlled trials published before September 2021 in the PubMed, Goggle scholar, and Web of Science databases were searched following the Preferred Reporting Items for Systematic Review and Meta-Analysis (PRISMA) guidelines.

**Supplementary Figure 2.** Number of studies meeting individual PEDro [Physiotherapy Evidence Database] criteria.
